# Effects of water and salt stress on seed germination and seedling growth of *Acanthus ebracteatus*

**DOI:** 10.3389/fpls.2026.1851398

**Published:** 2026-07-07

**Authors:** Lihao Tong, Tingwei Gao, Bing Yan, Wenai Liu

**Affiliations:** Guangxi Key Laboratory of Mangrove Conservation and Utilization, Guangxi Academy of Marine Sciences (Guangxi Mangrove Research Center), Guangxi Academy of Sciences, Beihai, China

**Keywords:** *Acanthus ebracteatus*, endangered mangrove, salinity, seed germination, seedling growth, water stress

## Abstract

**Introduction:**

Water deficit and salinity are primary abiotic filters shaping mangrove establishment. The endangered mangrove *Acanthus ebracteatus* Vahl possesses ecological and pharmacological importance, but its populations are declining. We investigated the combined effects of seed moisture content, salinity, and flooding duration on germination and seedling performance to determine environmental thresholds for conservation.

**Methods:**

Seed germination was tested under five moisture contents (17 (CK), 60, 41, 24, and 10%) and six salinities (0, 5, 10, 15, 25, and 35‰). Seedling growth and physiology were assessed under four flooding durations (1, 4, 8, and 12 h·d^-1^) at a background salinity of 5‰ and eight salinities (0, 5, 10, 15, 20, 25, 30, and 35‰) at a fixed flooding duration 4 h·d^-1^.

**Results:**

Decreasing seed moisture content from 60% to 10% progressively reduced germination percentage (from 93.3% to 24.4%), with naturally shed seeds (17% moisture content) exhibiting the poorest performance (17.8% germination). Salinity levels ≤15‰ maintained high germination percentages (>90%), whereas germination was severely inhibited at ≥25‰. Flooding duration did not affect seedling survival (100% in all treatments), but prolonged inundation (>4 h·d^-1^) reduced plant height and leaf area and altered antioxidant and osmotic regulation; the suitable daily flooding range was 1–4 h. Under salinity, seedling survival was 100% at 0–15‰, declined to 87.5% at 20‰, and dropped to 0% at ≥30‰. Growth and physiological functions were optimal at 0–15‰, with moderate salinity (5–10‰) promoting root biomass; salinity ≥20‰ caused growth inhibition and mortality.

**Conclusion:**

These findings demonstrate that *A. ebracteatus* exhibits multifactorial adaptation strategies to intertidal conditions, providing critical physiological benchmarks for conservation strategies targeting this threatened mangrove.

## 1 Introduction

Mangrove forests occupy intertidal zones where substrate water potential, salinity, and tidal flooding fluctuate substantially. These environmental gradients act as strong ecological filters for seed germination, seedling establishment, and subsequent population recruitment (Knight et al., 2008; Kimera et al., 2024; Yang et al., 2025). Salinity and tidal inundation are widely recognized as major abiotic filters affecting mangrove growth and establishment, especially during the early life stages (Liu et al., 2024; Kimera et al., 2024). Seed germination and seedling establishment are among the most vulnerable phases in the plant life cycle, because successful recruitment requires rapid water uptake, metabolic reactivation, radicle protrusion, and subsequent seedling survival under variable environmental conditions (Rajjou et al., 2012; Lu et al., 2022). Low water potential can restrict imbibition and delay metabolic recovery, whereas excessive salinity imposes osmotic stress, ion toxicity, and secondary oxidative stress, thereby inhibiting germination and early seedling growth (Hasanuzzaman et al., 2021; Chen et al., 2022). Recent studies on mangrove and coastal plants further indicate that high salinity can reduce seedling survival, biomass accumulation, and establishment success (Kimera et al., 2024; Liu et al., 2024). Flooding, another defining feature of intertidal habitats, limits oxygen diffusion in the root zone and may cause hypoxia or anoxia, thereby impairing aerobic respiration and carbon metabolism (Knight et al., 2013; Fagerstedt et al., 2024). In mangroves, responses to tidal flooding depend strongly on flooding duration and depth, and may vary among species and early life stages (Phandee et al., 2022; Yang et al., 2025). Although mangrove plants have evolved adaptive traits such as salt regulation, specialized root structures, and waterlogging-tolerance mechanisms, the combined effects of water deficit, salinity, and flooding on germination and seedling establishment remain insufficiently understood for many rare mangrove species (Ye et al., 2003; Yuan et al., 2016).

*Acanthus ebracteatus* Vahl (Acanthaceae) is a true mangrove species with important ecological and medicinal value. It usually occurs along the landward edge of mangrove forests or tidal channels and represents an ecologically and conservation-relevant component of subtropical mangrove communities (Chen et al., 2025). Pharmacological studies have shown that *A. ebracteatus* contains bioactive compounds with antioxidant, anti-inflammatory, anti-aging, neuroprotective, wound-healing, and photoprotective potential (Prasansuklab and Tencomnao, 2018; Olatunji et al., 2022; Kanlayavattanakul et al., 2024). Despite these ecological and medicinal values, *A. ebracteatus* is endangered in China and has a high conservation priority (Fang et al., 2024; Chen et al., 2025; Ni et al., 2025). In China, *A. ebracteatus* is naturally distributed in Guangxi, Guangdong, and Hainan, with field surveys indicating that its remaining populations are small, fragmented, and vulnerable to habitat degradation, particularly in Guangxi (Huang et al., 2020; Lin et al., 2022; Chen et al., 2025).

Our preliminary field observations suggest that recruitment failure of *A. ebracteatus* may be associated with post-dispersal seed desiccation and unsuitable salinity and inundation regimes during early establishment. The seeds are shed with high moisture content and appear sensitive to water loss, whereas seedlings must subsequently tolerate periodic flooding and salinity stress in intertidal habitats. Similar regeneration bottlenecks have been reported in mangrove and coastal plant recruitment, where salinity, water availability, and microhabitat conditions jointly constrain early establishment (Kimera et al., 2024; Yang et al., 2025). However, quantitative information on the critical seed moisture content, salinity thresholds, and flooding durations that permit successful germination and early seedling growth remains limited. Such knowledge is essential for defining the ecological regeneration niche of this species and for developing evidence-based propagation and restoration protocols.

We therefore conducted a factorial experiment to: (i) determine the critical seed moisture content and salinity range for germination; (ii) evaluate seedling responses to simulated tidal flooding and salinity; and (iii) delineate the optimal environmental envelope for germination and early establishment of this rare mangrove species. By linking germination, growth, survival, and physiological indicators, this study provides quantitative guidance for *ex situ* propagation, field introduction, and habitat restoration of *A. ebracteatus*.

## 2 Materials and methods

### 2.1 Experimental materials

**Figure 1:**
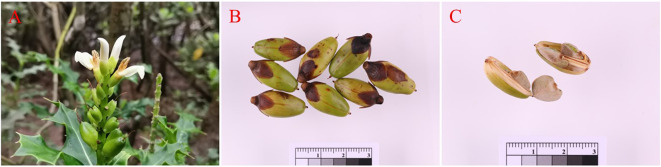
Fruit and seed morphology of *A*. *ebracteatus*. **(A)** Habit and branch of *A*. *ebracteatus*; **(B)** mature fruit; **(C)** seed morphology.

Seeds of *A. ebracteatus* were collected in July 2023 from a wild population at Bailong Peninsula, Fangchenggang City, Guangxi, China (21°30′59.30″ N, 108°13′36.09″ E; **Figure 1**). Both naturally dehulled seeds (mature fruits dehisced on the plant) and manually dehulled seeds (mature but indehiscent fruits collected on the same day and opened manually) were obtained from the same maternal individuals to minimize genetic variation. Intact and plump seeds were selected for the germination experiments.

Seedlings for stress experiments were raised from the same seed batch in July 2023. Germinated seeds were transplanted into individual pots containing peat soil and cultivated in a glasshouse under natural light with daily watering using freshwater. After three months, the seedlings reached a height of 12.98 ± 0.75 cm and a basal diameter of 3.55 ± 0.30 mm, at which stage they were used for the experiments.

### 2.2 Seed germination experiment

#### 2.2.1 Experimental design for seed moisture content

The room-temperature silica gel desiccation method was employed. Manually dehulled seeds were placed in nylon mesh bags and embedded in silica gel within desiccators. Dehydration was performed at room temperature (25 °C) for 24 h. Seeds with varying moisture contents were prepared by adjusting the weight ratio of silica gel to seeds. Using the moisture content of naturally dehulled seeds (17%) as the control (CK), four treatment groups of manually dehulled seeds were established: 60% (without silica gel desiccation), 41%, 24%, and 10% moisture content. Each treatment was replicated three times, with 15 seeds per replicate.

Seeds with different moisture contents were soaked in freshwater for 24 h and then placed in Petri dishes lined with moist filter paper for a 7-d germination experiment. Germinated seeds (defined by radicle emergence through the seed coat) were observed and counted daily. Each Petri dish was replenished daily with 5 mL of freshwater to maintain moist conditions throughout the experiment.

#### 2.2.2 Experimental design for salinity stress in seeds

Uniformly sized and plump manually dehulled seeds were selected as experimental materials. Six salinity treatments were established: 0, 5, 10, 15, 25, and 35‰. The salinity levels were selected based on historical salinity data from the surrounding coastal waters, aiming to encompass the widest possible range of salinities (0–35‰) that seeds and seedlings may encounter. Each treatment was replicated three times, with 15 seeds per replicate. Artificial seawater, prepared by adding sea salt to tap water to achieve target salinity levels, was used in the experiment.

Seeds were soaked in the artificial seawater at the corresponding salinity levels for 24 h and then placed in Petri dishes lined with moist filter paper for a 7-d germination experiment. The number of germinated seeds was observed and counted daily. Each Petri dish was replenished daily with 5 mL of seawater at the corresponding salinity to keep the filter paper moist. Artificial seawater was prepared by dissolving commercial sea salt (China National Salt Industry Corporation, Tianjin, China) in tap water, with salinity confirmed using a calibrated conductivity meter (Model AZ8373, AZ Instrument, Taichung, Taiwan).

### 2.3 Experimental design for flooding and salinity stress in seedlings

**Figure 2:**
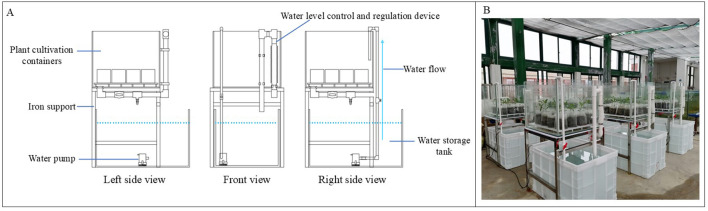
Artificial tidal simulation system. **(A)** Structural diagram showing the arrangement of the water storage tank, plant cultivation containers, and water pump; **(B)** actual view of the experimental setup in the greenhouse.

An artificial tidal simulation system was established using water storage tanks, plant cultivation containers, timers, water pumps, and a shading system (**Figure 2**). The flooding level and duration for each treatment were uniformly controlled via an automatic control device. Peat soil was used as the planting substrate, and the flooding level was set to completely submerge the seedlings throughout the experimental period. All seedling experiments were conducted from October to December 2023 in a glasshouse. During this period, the maximum temperature was 43.5 °C, the minimum temperature was 9.0 °C, the average temperature was 24.0 °C, the maximum daytime light intensity (7:00–18:00) was 28,109 Lux, and the average light intensity was 3,635 Lux. These temperature fluctuations reflect natural seasonal variation in a non-environmentally controlled nursery glasshouse; to minimize potential confounding effects, treatment pots were fully randomized within the glasshouse, and temperature was recorded daily. Throughout the experiments, appropriate amounts of freshwater or sea salt were added daily to maintain stable water levels and target salinities.

Four flooding duration treatments were established: 1, 4, 8, and 12 h·d^-1^, representing a gradient from high-intertidal to low-intertidal inundation regimes and simulating diurnal tides with a 24-h tidal cycle. Each treatment consisted of four replicates, with four seedlings per replicate, totaling 64 seedlings. A background salinity of 5‰ was used throughout this experiment, as this value represents the low-brackish salinity conditions observed in the local estuary during the rainy season, when germination and early establishment are most likely to occur, and was confirmed in preliminary tests to support favorable seedling growth.

Eight salinity treatments were established: 0, 5, 10, 15, 20, 25, 30, and 35‰. These values were selected based on long-term historical salinity data from the coastal waters near the study area, encompassing the full range that *A. ebracteatus* propagules may encounter. Artificial seawater was prepared by dissolving commercial sea salt (China National Salt Industry Corporation, Tianjin, China) in tap water, and salinity was verified for each batch using a calibrated conductivity meter (Model AZ8373, AZ Instrument, Taichung, Taiwan). Each treatment consisted of four replicates, with four seedlings per replicate, totaling 128 seedlings. All treatments were subjected to a fixed daily flooding duration of 4 h, representing the mid-intertidal inundation regime typical of the species’ natural habitat and allowing the independent effect of salinity to be assessed under ecologically realistic flooding conditions.

### 2.4 Measurement of indicators

#### 2.4.1 Germination parameters


$$\text{Germination percentage}=\frac{Number\;of\;normally\;germinated\;seeds}{Total\;number\;of\;seeds}\times 100\%$$

$$\text{Germination index}=\sum (G_{t}/T_{t})$$

where *G_t_* is the number of seeds germinated on day *t*, and *T_t_* is the number of days.


Mean germination time=∑(Ti×Ni)/∑Ni

where *Ni* is the number of newly germinated seeds at time *Ti*.


$$\text{Germination energy}=\frac{Number\;of\;germinated\;seeds\;at\;the\;maximum\;germination\;day}{Total\;number\;of\;seeds}\times 100\%$$

#### 2.4.2 Seedling growth and physiological indicators

Survival rate, plant height, basal diameter, leaf number, and individual leaf area (Yaxin-1241 portable leaf area meter) were measured at the end of the experiment. For biomass determination, four seedlings per treatment were separated into above- and below-ground parts, oven-dried (105 °C for 20 min, then 80 °C to constant weight), and weighed (0.001 g precision).

Physiological and biochemical analyses were performed using the second pair of fully expanded mature leaves. Fresh leaves were rinsed with tap water, gently surface-dried, and stored at −80 °C until analysis. All assays were conducted using commercial kits from Suzhou Grace Biotechnology Co., Ltd. (Suzhou, China) according to the manufacturer’s instructions. Approximately 0.10 g of fresh leaf tissue was used for each assay. The assays followed established spectrophotometric or colorimetric principles, whereas the specific extraction buffers, reaction volumes, wavelengths, calibration equations, and calculation procedures followed the kit protocols.

For antioxidant enzyme assays, fresh leaf tissue was homogenized with 1.0 mL of ice-cold extraction buffer and centrifuged at 12,000 rpm for 10 min at 4 °C. Superoxide dismutase (SOD) activity was determined using the WST-8 method based on the inhibition of water-soluble tetrazolium salt reduction by superoxide anions generated in the xanthine–xanthine oxidase system (Ukeda et al., 1999). Absorbance was measured at 450 nm, and one unit of SOD activity was defined as the amount of enzyme causing 50% inhibition of WST-8 reduction. Catalase (CAT) activity was measured using a colorimetric assay based on H_2_O_2_ decomposition, modified from the classical catalase assay principle (Aebi, 1984). The remaining H_2_O_2_ reacted with a chromogenic reagent and was detected at 510 nm. One unit of CAT activity was defined as the amount of enzyme decomposing 1 μmol H_2_O_2_ min^-^¹ at 25 °C. Peroxidase (POD) activity was determined using the guaiacol oxidation method (Chance and Maehly, 1955). The increase in absorbance at 470 nm was recorded within 1 min. SOD, CAT, and POD activities were expressed as U·g^-1^ fresh weight (FW).

Malondialdehyde (MDA) content was determined using the thiobarbituric acid reactive substances method (Heath and Packer, 1968). The reaction mixture was heated at 90–95 °C for 30 min, and absorbance was measured at 532 and 600 nm. MDA content was calculated from A_532_ − A_600_ and expressed as nmol·g^-1^ FW. Free proline content was measured using the acid ninhydrin colorimetric method (Bates et al., 1973). The extract was reacted at 95 °C for 30 min, absorbance was measured at 520 nm, and proline content was expressed as μg·g^-1^ FW.

Soluble protein content was determined using a modified Coomassie brilliant blue G-250 method (Bradford, 1976). According to the kit protocol, absorbance was measured at 600 nm, and soluble protein content was expressed as mg·g^-1^ FW. Soluble sugar content was determined using the anthrone colorimetric method (Yemm and Willis, 1954). Fresh tissue was extracted with 80% ethanol, the reaction mixture was heated at 95–100 °C for 10 min, absorbance was measured at 620 nm, and soluble sugar content was expressed as mg·g^-1^ FW.

Photosynthetic pigments were extracted from approximately 0.10 g of fresh leaf tissue using 95% ethanol extraction buffer under dark or dim-light conditions. The extract was adjusted to 10 mL and kept in darkness until the tissue residue was completely bleached. Absorbance was measured at 665 and 649 nm. Chlorophyll *a* and chlorophyll *b* contents were calculated according to the equations provided in the kit protocol, based on the general spectrophotometric principles of chlorophyll determination in organic solvent extracts (Lichtenthaler, 1987; Wellburn, 1994), and expressed as mg·g^-1^ FW.

### 2.5 Statistical analysis

Data were processed using Microsoft Excel 2019 and SPSS 18.0. Normality was verified with the Shapiro−Wilk test and homogeneity of variances was assessed with Levene’s test. One−way ANOVA was applied to each experiment, and multiple comparisons among treatments were performed using Tukey’s HSD test (*P* < 0.05). Figures were generated with Origin 2021. The experiments were independent single−factor designs; future studies will employ factorial designs to explore interactions among water availability, salinity, and flooding.

## 3 Results

### 3.1 Effect of seed moisture content on seed germination

**Figure 3:**
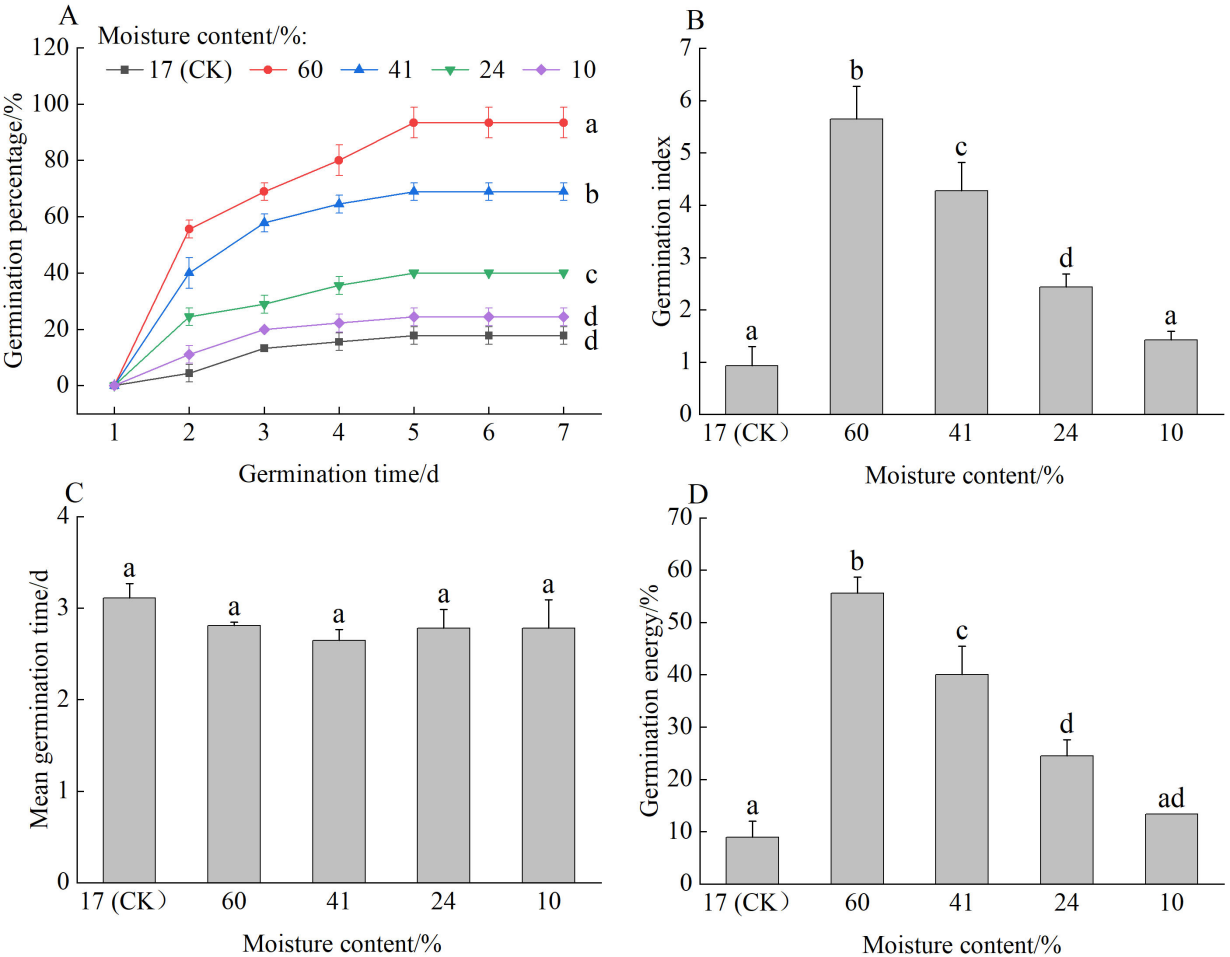
Effects of seed moisture content on seed germination of *A*. *ebracteatus*. **(A)** Germination percentage (%); **(B)** germination index; **(C)** mean germination time (d); **(D)** germination energy (%). Values represent mean ± SD (n = 3 replicates per treatment). Different letters above the bars indicate significant differences among treatments (*P* < 0.05).

Seed moisture content significantly affected germination percentage, germination index, and germination energy (P < 0.05; **Figure 3**). All treatments initiated germination on day 2. Naturally dehulled seeds (17% moisture content) exhibited the lowest values for all parameters, which were significantly lower than those of manually dehulled seeds with 60%, 41%, and 24% moisture content. Among manually dehulled seeds, germination declined monotonically with decreasing moisture content; the 24% and 10% groups were significantly lower than the 41% and 60% groups. Mean germination time did not differ significantly among treatments.

### 3.2 Effect of salinity on seed germination

**Figure 4:**
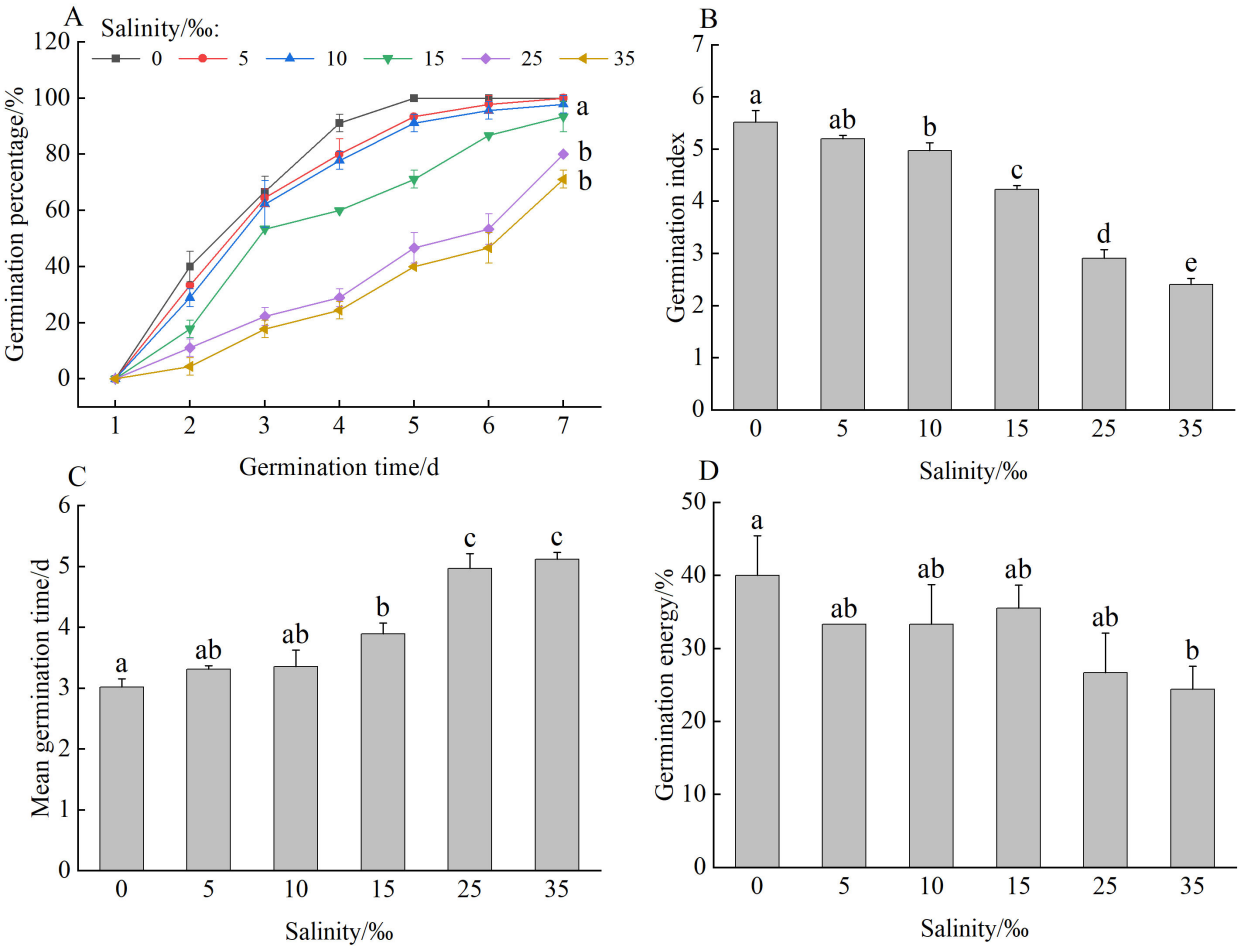
Effects of salinity on seed germination of *A*. *ebracteatus*. **(A)** Germination percentage (%); **(B)** germination index; **(C)** mean germination time (d); **(D)** germination energy (%). Values represent mean ± SD (n = 3 replicates per treatment). Different letters above the bars indicate significant differences among treatments (*P* < 0.05).

Salinity significantly affected all germination parameters (*P* < 0.05; **Figure 4**). Germination percentage was highest (100%) at 0 and 5‰ and remained above 90% at 10 and 15‰, whereas it dropped markedly at 25 and 35‰. A similar threshold was observed at 15‰ for both the germination index and germination energy, beyond which values decreased significantly. Mean germination time was significantly prolonged at 25 and 35‰ compared with the 0–15‰ treatments (*P* < 0.05).

### 3.3 Effect of flooding duration on seedling growth and physiology

**Figure 5:**
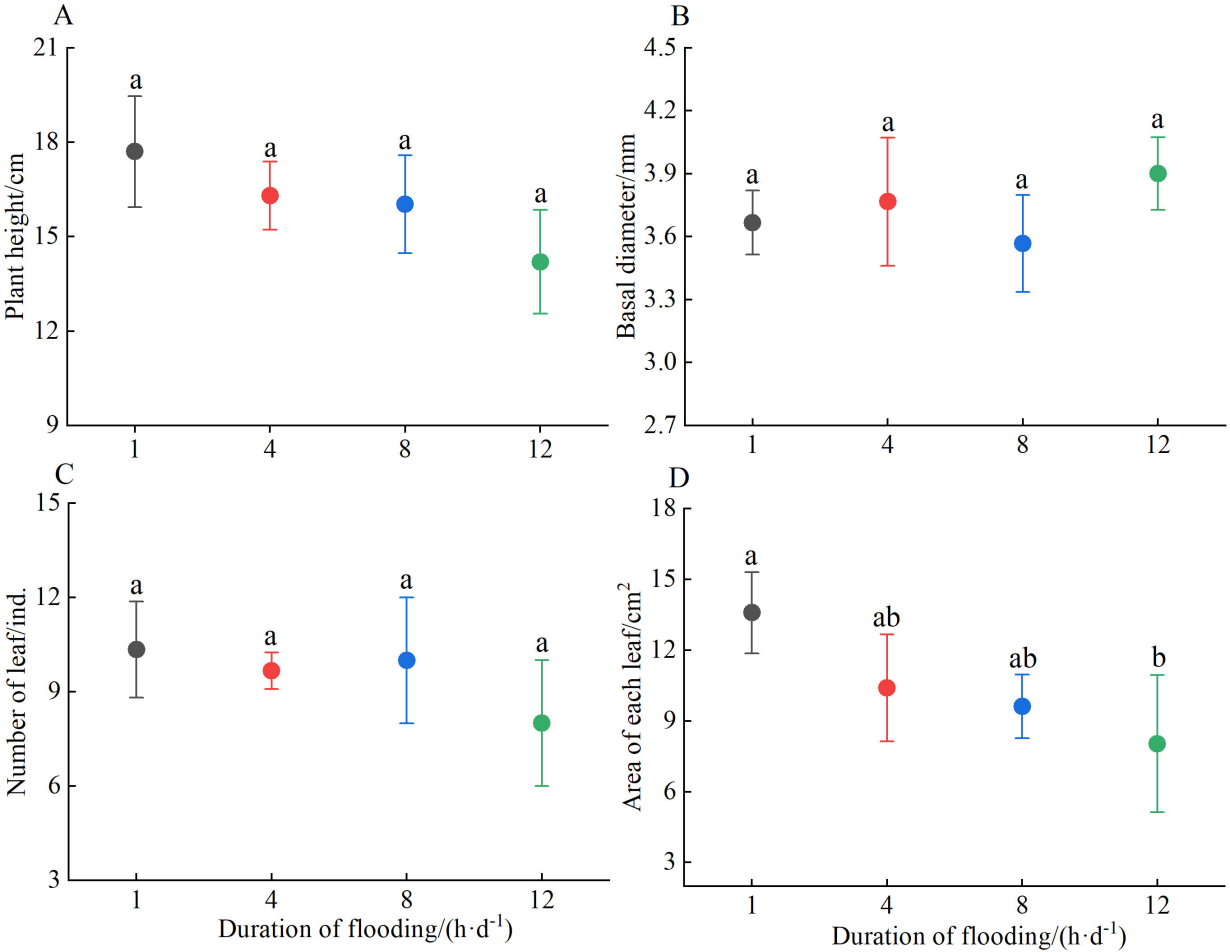
Effect of flooding duration on growth indicators of *A*. *ebracteatus* seedlings. **(A)** Plant height (cm); **(B)** basal diameter (mm); **(C)** leaf number; **(D)** individual leaf area (cm²). Values represent mean ± SD (n = 3 replicates per treatment). Different letters above the bars indicate significant differences among treatments (*P* < 0.05).

Survival rate was 100% across all flooding treatments. Plant height, basal diameter, and leaf number did not differ significantly among treatments. Individual leaf area was significantly greater in the 1 h·d^-1^ group than in the 12 h·d^-1^ group (*P* < 0.05), while no significant differences were observed among the 1, 4, and 8 h·d^-1^ groups (**Figure 5**).

**Figure 6:**
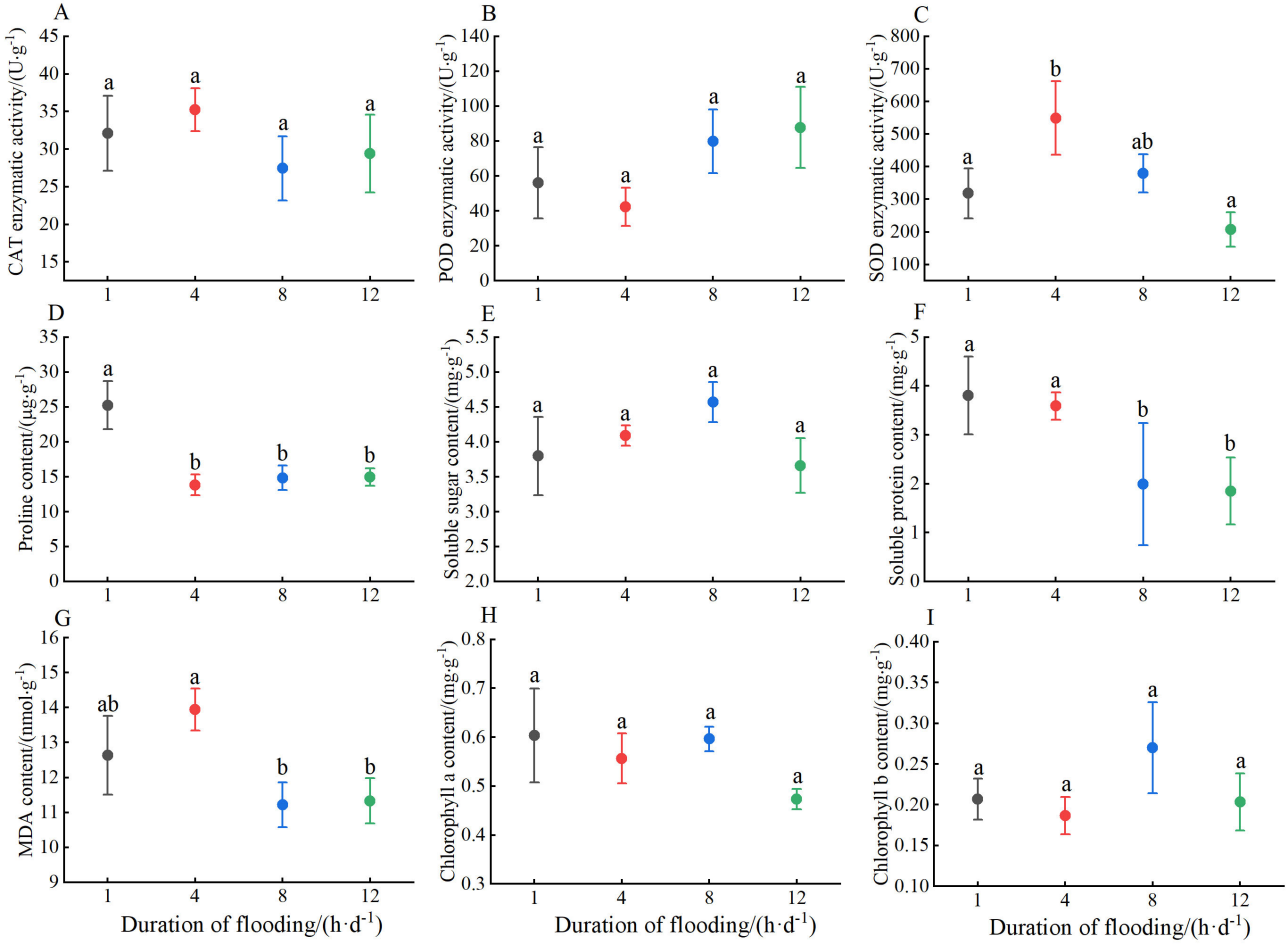
Effect of flooding duration on antioxidant enzymes, osmoregulatory substances, and photosynthetic pigments in the leaves of *A*. *ebracteatus* seedlings. **(A)** CAT activity (U·g^-^¹ FW); **(B)** POD activity (U·g^-^¹ FW); **(C)** SOD activity (U·g^-^¹ FW); **(D)** proline content (μg·g^-^¹ FW); **(E)** soluble sugar content (mg·g^-^¹ FW); **(F)** soluble protein content (mg·g^-^¹ FW); **(G)** MDA content (nmol·g^-^¹ FW); **(H)** chlorophyll *a* content (mg·g^-^¹ FW); **(I)** chlorophyll *b* content (mg·g^-^¹ FW). Values represent mean ± SD (n = 3 replicates per treatment). Different letters above the bars indicate significant differences among treatments (*P* < 0.05). CAT, catalase; POD, peroxidase; SOD, superoxide dismutase; MDA, malondialdehyde; FW, fresh weight.

CAT and POD activities, soluble sugar content, and chlorophyll *a* and *b* concentrations did not differ significantly among flooding treatments (*P* > 0.05). Proline and soluble protein contents decreased with prolonged flooding duration. Proline content was significantly higher in the 1 h·d^-1^ treatment than in all other treatments (*P* < 0.05), and soluble protein content was significantly greater at 1 and 4 h·d^-1^ than at 8 and 12 h·d^-1^. Both SOD activity and MDA content exhibited a unimodal pattern, peaking at 4 h·d^-1^ and declining thereafter (**Figure 6**).

### 3.4 Effect of salinity on seedling growth and physiology

**Figure 7:**
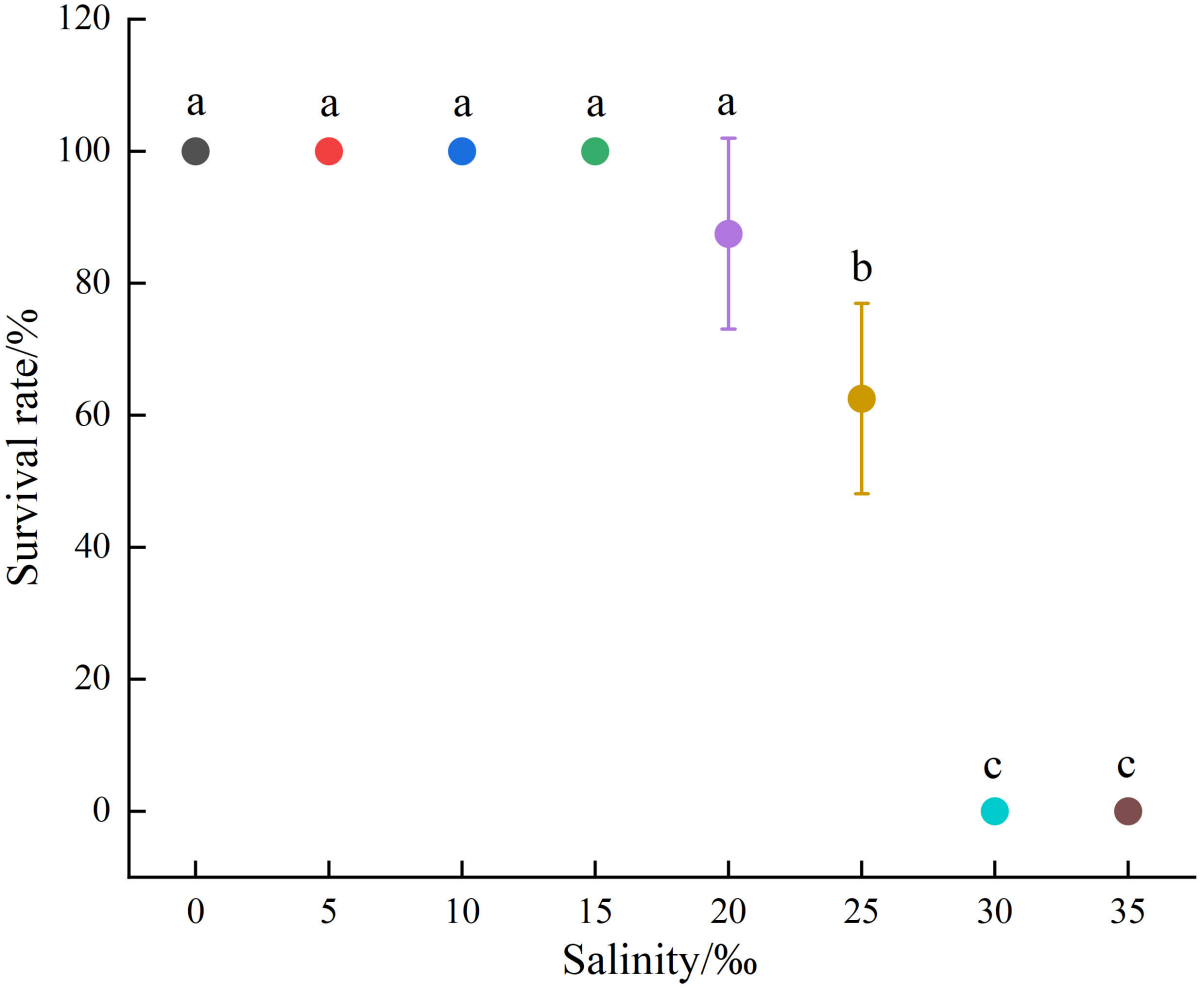
Effects of salinity on the survival rate (%) of *A*. *ebracteatus* seedlings. Values represent mean ± SD (n = 3 replicates per treatment). Different letters above the bars indicate significant differences among treatments (*P* < 0.05).

**Figure 8:**
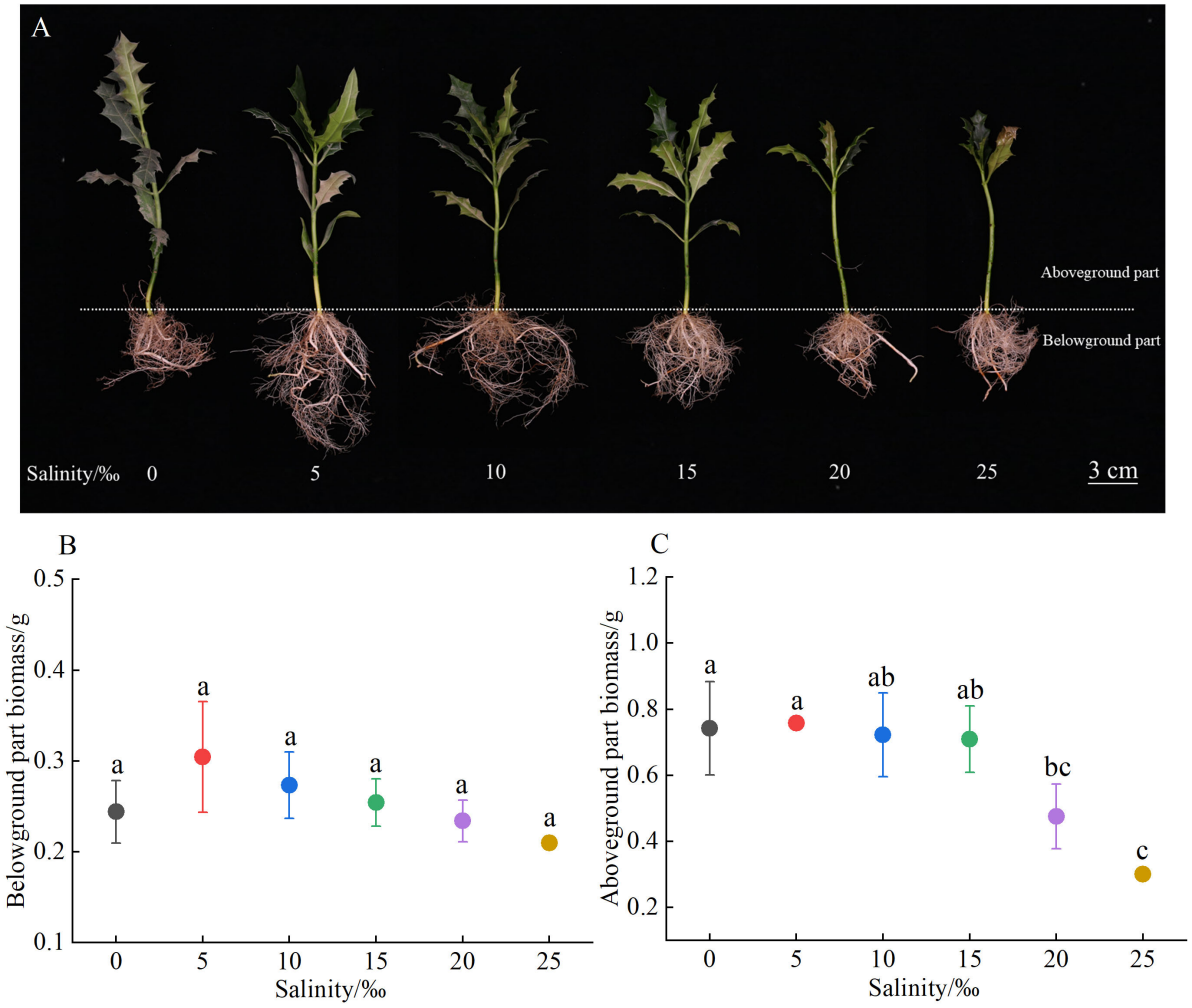
Effects of salinity on biomass accumulation in *A*. *ebracteatus* seedlings. **(A)** Whole-plant morphology of representative seedlings under different salinity treatments; **(B)** belowground biomass (g dry weight); **(C)** aboveground biomass (g dry weight). Values represent mean ± SD (n = 3 replicates per treatment). For each biomass component, different letters above the bars indicate significant differences among treatments (*P* < 0.05).

**Figure 9:**
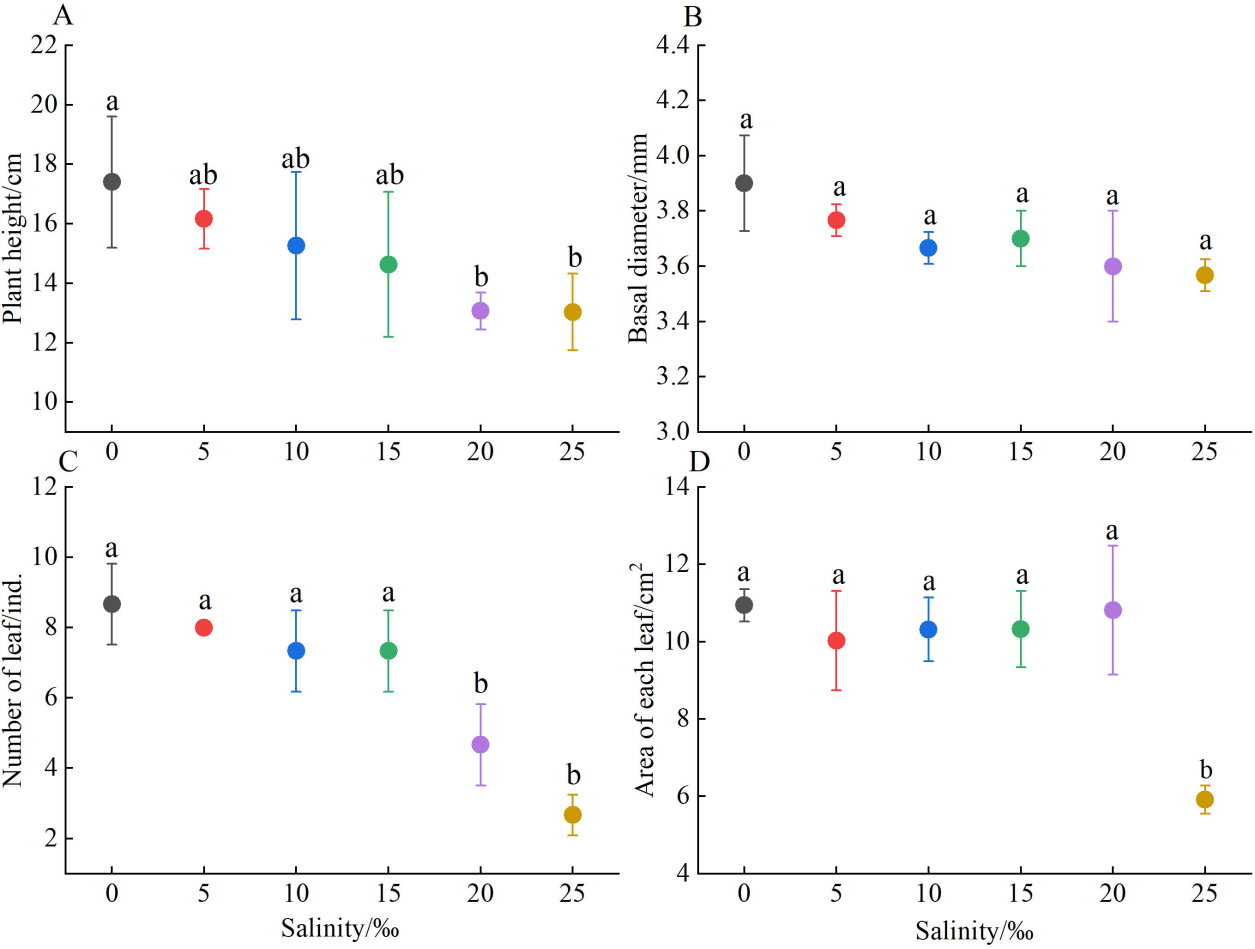
Effect of salinity on growth indicators of *A*. *ebracteatus* seedlings. **(A)** Plant height (cm); **(B)** basal diameter (mm); **(C)** leaf number; **(D)** individual leaf area (cm²). Values represent mean ± SD (n = 3 replicates per treatment). Different letters above the bars indicate significant differences among treatments (*P* < 0.05).

Survival was 100% at 0–15‰, declined to 87.5% at 20‰, and reached 0% at ≥30‰ (**Figure 7**). Root development was poorest at 0‰ and most vigorous at 5–10‰. Biomass accumulation was stimulated at low salinity (0–15‰) and inhibited at higher salinities (**Figure 8**). Plant height did not differ significantly among the 0–15‰ treatments (*P* > 0.05) but decreased markedly at ≥20‰. Basal diameter did not differ significantly among any salinity treatments (*P* > 0.05). Leaf number was significantly greater at 0–15‰ than at 20–25‰ (*P* < 0.05), with severe leaf abscission observed at ≥20‰ (**Figure 9**). Individual leaf area was significantly reduced at 25‰ compared with all other treatments (*P* < 0.05).

**Figure 10:**
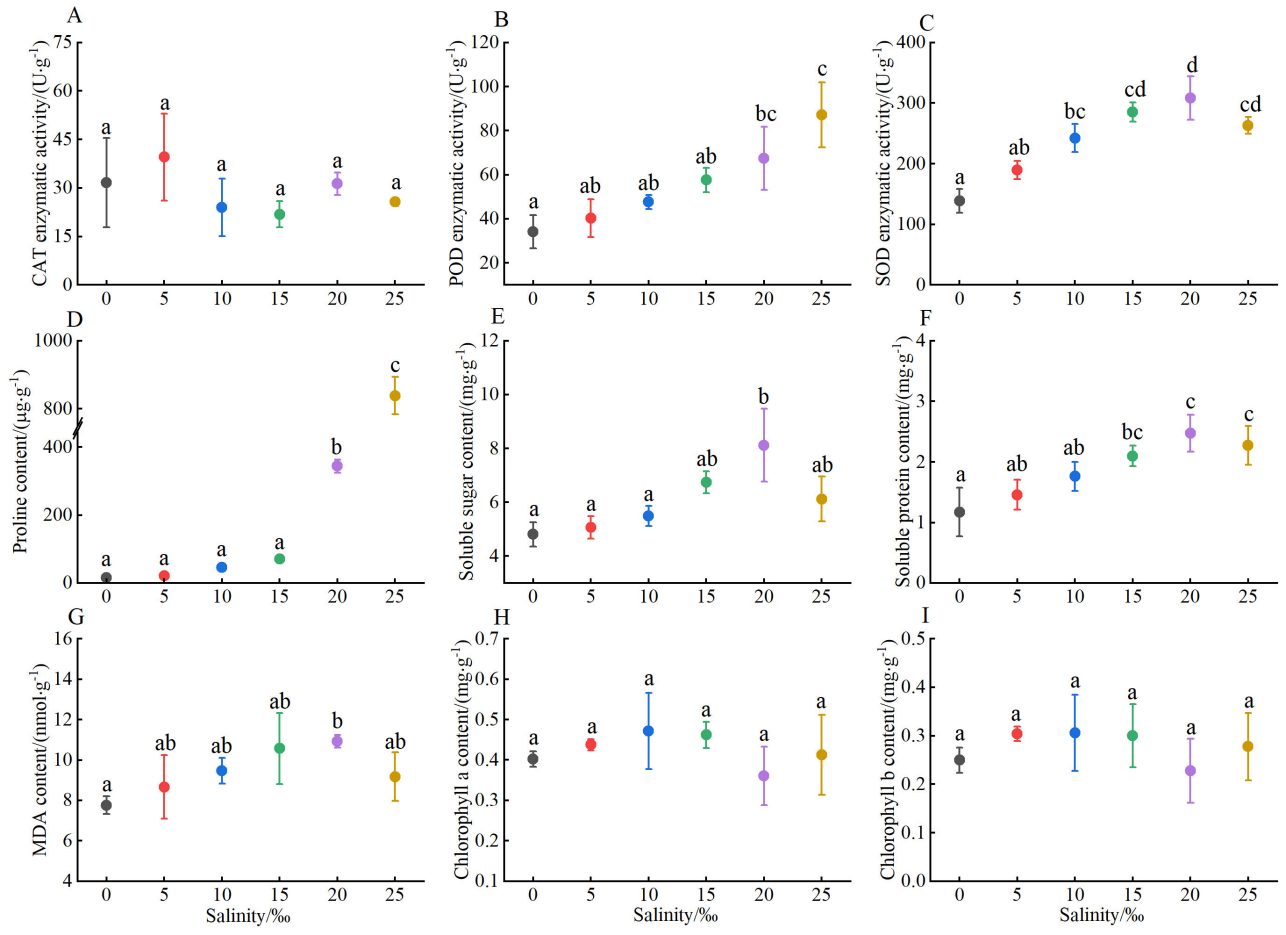
Effect of salinity on antioxidant enzymes, osmoregulatory substances, and photosynthetic pigments in the leaves of *A*. *ebracteatus* seedlings. **(A)** CAT activity (U·g^-^¹ FW); **(B)** POD activity (U·g^-^¹ FW); **(C)** SOD activity (U·g^-^¹ FW); **(D)** proline content (μg·g^-^¹ FW); **(E)** soluble sugar content (mg·g^-^¹ FW); **(F)** soluble protein content (mg·g^-^¹ FW); **(G)** MDA content (nmol·g^-^¹ FW); **(H)** chlorophyll *a* content (mg·g^-^¹ FW); **(I)** chlorophyll *b* content (mg·g^-^¹ FW). Values represent mean ± SD (n = 3 replicates per treatment). Different letters above the bars indicate significant differences among treatments (*P* < 0.05). CAT, catalase; POD, peroxidase; SOD, superoxide dismutase; MDA, malondialdehyde; FW, fresh weight.

CAT activity and chlorophyll *a* and *b* concentrations did not differ significantly among salinity treatments (*P* > 0.05). POD activity increased progressively with increasing salinity (*P* < 0.05). Proline content did not differ significantly among the 0–15‰ treatments but rose sharply at 20‰ and 25‰, where it was significantly higher than that in the 0–15‰ groups (*P* < 0.05). SOD activity, soluble sugar content, soluble protein content, and MDA content all exhibited a unimodal pattern, peaking at 20‰ and declining thereafter (**Figure 10**).

## 4 Discussion

This study shows that seed moisture content, salinity, and flooding duration jointly define the regeneration niche of *A. ebracteatus*. The combination of seed desiccation sensitivity, a relatively narrow salinity tolerance during germination, and a preference for moderate inundation provides a plausible explanation for the restricted recruitment of this species in low-salinity, mid- to high-intertidal microhabitats. Similar stage-specific environmental filters have been reported in mangrove and coastal plant regeneration, where salinity, water availability, and tidal conditions strongly affect early establishment (Kimera et al., 2024; Yang et al., 2025).

### 4.1 Seed moisture content and seed germination: desiccation sensitivity

Seeds of *A. ebracteatus* are shed with high moisture content and germinate rapidly if they encounter a moist substrate. However, field observations indicate that litter often prevents seeds from reaching the soil, leaving them exposed to air−drying. Our results show that germination percentage, germination index, and germination energy declined progressively as seed moisture content decreased from 60% to 10%. Notably, naturally dehulled seeds (17% moisture content) performed significantly worse than manually dehulled seeds with similar or even lower moisture content. This contrast suggests that post-dispersal exposure under field conditions may cause physiological damage beyond simple water-content reduction, possibly through rapid dehydration, temperature fluctuations, microbial attack, or mechanical injury.

This extreme sensitivity to water loss is consistent with the behavior of recalcitrant seeds, which lack the ability to maintain cellular integrity under desiccation (Berjak and Pammenter, 2007). However, we note that a definitive classification would require determination of a desiccation tolerance curve and critical water content, which were beyond the scope of this study. The poor germination of naturally dehulled seeds also suggests that microhabitat desiccation is a major contributor to the regeneration failure observed in wild populations (Berjak and Pammenter, 2007; Yang et al., 2025). Similar negative effects of water stress on germination have been reported for other aromatic and herbaceous species such as *Thymus satureioides*, *Elymus nutans*, and *Lavandula stoechas* (Benadjaoud et al., 2022; Long et al., 2023; Oublid et al., 2024), though the underlying mechanisms may differ given the contrasting ecological contexts.

### 4.2 Salinity tolerance during seed germination

Salt stress reduced all germination parameters of *A. ebracteatus* and lengthened mean germination time. The inhibitory effects of salinity on seed germination are well documented across diverse taxa, from crops to halophytes (Ajmal Khan et al., 2006; Jamil et al., 2006; Santo et al., 2017). At the physiological level, salt−induced suppression of gibberellin biosynthesis and promotion of abscisic acid accumulation have been shown to delay or inhibit germination in rice and soybean (Liu et al., 2018; Shu et al., 2017). For *A. ebracteatus*, germination remained robust (≥90%) up to 15‰ but declined sharply at 25‰. This threshold (15‰) is relatively low compared with that of some other mangrove associates; for example, certain coastal halophytes show no germination reduction until salinity exceeds 25–30‰ (Wijayasinghe et al., 2018; Chen et al., 2022; Kimera et al., 2024). The comparatively narrow salinity tolerance of *A. ebracteatus* may be linked to its preference for upper−estuarine, low−salinity microhabitats (Huang et al., 2020; Lin et al., 2022; Chen et al., 2025). At elevated salinities, osmotic inhibition of water uptake and ion toxicity likely become primary constraints on germination, whereas species-specific salt exclusion or secretion capacity may modulate tolerance among mangroves (Jamil et al., 2006; Yuan et al., 2016; Hasanuzzaman et al., 2021). Thus, the germination stage appears to act as a strong ecological filter, restricting recruitment to periods and locations of low salinity.

### 4.3 Flooding duration: physiological responses and growth constraints

All *A. ebracteatus* seedlings survived irrespective of daily flooding duration (1–12 h), confirming strong waterlogging tolerance. However, prolonged inundation imposed sub−lethal growth constraints: individual leaf area was significantly reduced in the 12 h·d^-1^ treatment compared with the 1 h·d^-1^ treatment, whereas plant height, basal diameter, and leaf number were not significantly affected. This pattern differs from that of low−intertidal species such as *Avicennia marina*, which shows optimal growth at 8–12 h of daily flooding (Liao et al., 2010b). The lower flooding optimum of *A. ebracteatus* aligns with its predominant occurrence in mid− to high−intertidal zones, where inundation periods are naturally shorter.

Regarding oxidative stress markers, CAT activity and soluble sugar content did not change significantly across flooding treatments. SOD activity and MDA content exhibited parallel unimodal patterns, both peaking at 4 h·d^-1^ and declining at longer durations. The simultaneous decrease in SOD and MDA under extended flooding suggests that lipid peroxidation was not exacerbated and, therefore, that severe oxidative injury did not occur (Tang et al., 2010; Phandee et al., 2022; Liu et al., 2023). This interpretation is reinforced by the 100% survival rate. It is possible that other antioxidant components—perhaps POD, which showed a tendency towards higher activity under prolonged flooding although the difference was not significant—helped maintain redox homeostasis (Phandee et al., 2022; Liu et al., 2023; Wang et al., 2019). The decline in proline and soluble protein contents with increasing flooding duration may reflect a reduced demand for osmotic adjustment once metabolic activity is down−regulated under chronic hypoxia (Knight et al., 2013; Liu et al., 2023; Fagerstedt et al., 2024). Overall, the physiological data indicate that *A. ebracteatus* seedlings tolerate a wide range of flooding durations, but growth performance was most favorable when daily submergence was limited to 1–4 h. This range is therefore considered suitable for the species.

### 4.4 Salinity thresholds for seedling establishment

Salinity exerted a strong control on seedling performance. Survival was 100% up to 15‰, declined to 87.5% at 20‰, and dropped to 0% at ≥ 30‰. Growth parameters generally displayed a low−salinity stimulation–high−salinity inhibition pattern. Root development was most vigorous at 5–10‰, a phenomenon also reported for *Bruguiera gymnorrhiza* and *Rhizophora stylosa* (Clough, 1984; Wang and Lin, 1999). Above 15‰, plant height decreased markedly, leaf abscission was observed at ≥ 20‰, and leaf area was significantly reduced at 25‰. These responses mirror the salinity−induced growth suppression documented in other mangrove species such as *B. sexangula* var. *rhynchopetala* and *Kandelia obovata* (Liao et al., 2010a; You, 2015; Liu et al., 2024).

At the biochemical level, POD activity rose progressively with salinity, whereas CAT activity and photosynthetic pigment concentrations remained unchanged. SOD activity, soluble sugar content, soluble protein content, and MDA content all peaked at 20‰ and then declined. The concurrent decline in these parameters at 25‰ suggests that the antioxidant machinery and osmotic adjustment capacity become overwhelmed beyond 20‰, leading to membrane damage and metabolic failure (Parida et al., 2004; Hasanuzzaman et al., 2021). The marked increase in proline at 20–25‰ may reflect enhanced osmotic adjustment under high salinity, although additional ion-balance and cellular damage indicators are needed to confirm this mechanism (Takemura et al., 2000; Parida et al., 2004; Hasanuzzaman et al., 2021). Taken together, these data identify 20‰ as a critical salinity threshold: *A. ebracteatus* seedlings can survive and maintain physiological function at 20‰, but growth is already suppressed, and survival drops sharply above this level. In contrast, *B. sexangula* var. *rhynchopetala* survives up to 25‰ (Liao et al., 2010a), indicating interspecific variation in salt tolerance among mangrove species (Clough, 1984; Patel et al., 2010). The relatively low salt tolerance of *A. ebracteatus* is consistent with its ecological niche in low−salinity estuarine habitats and underscores its vulnerability to salinity increases caused by reduced freshwater input or sea−level rise.

### 4.5 Limitations and future directions

Several limitations of this study warrant mention. The experiments were conducted under greenhouse conditions with natural temperature fluctuations (9–43.5 °C), which, although randomized across treatments, may have introduced uncontrolled variation. The comparison between naturally dehulled and manually dehulled seeds, despite using the same maternal individuals, cannot fully exclude differences in post−dispersal age and micro−environment. Furthermore, we assessed only short−term physiological responses; long−term survival, reproductive output, and performance under combined tidal and salinity dynamics remain to be tested. The classification of *A. ebracteatus* seeds as recalcitrant is preliminary; a formal desiccation tolerance curve is needed (Berjak and Pammenter, 2007). Future work should incorporate field transplant experiments, multi−season monitoring, and molecular analyses (e.g., transcriptomics of stress−responsive genes) to elucidate the mechanisms underlying the species’ environmental thresholds (Liu et al., 2023; Ni et al., 2025). Such information will be essential for designing effective *ex situ* conservation and habitat restoration programs (Andivia et al., 2021; Jarrar et al., 2023; Yang et al., 2025).

## 5 Conclusion

This study identifies the critical environmental thresholds that define the regeneration niche of the endangered mangrove *A. ebracteatus*. Seed germination depends on the maintenance of high seed moisture content (≥ 41% for manually dehulled seeds), with desiccation rapidly impairing viability. Salinity levels ≤ 15‰ supported both robust germination and 100% seedling survival, whereas salinity ≥ 20‰ progressively inhibited growth and became lethal at ≥ 30‰. Seedlings displayed strong tolerance to waterlogging, with a suitable daily inundation range of 1–4 h. These findings highlight a narrow regeneration window that is consistent with the species’ restricted distribution in low−salinity, mid− to high−intertidal microhabitats. The identified thresholds provide quantitative guidance for selecting restoration sites and optimizing *ex situ* propagation protocols. Although the study was conducted under greenhouse conditions and assessed short−term responses, the results offer a physiological baseline for future field−based validation and long−term monitoring, which are essential for the conservation of this threatened mangrove.

## Data Availability

The original contributions presented in the study are included in the article/supplementary material. Further inquiries can be directed to the corresponding author.
